# Acquisition and transmission of two ‘*Candidatus* Liberibacter solanacearum’ haplotypes by the tomato psyllid *Bactericera cockerelli*

**DOI:** 10.1038/s41598-020-70795-4

**Published:** 2020-08-19

**Authors:** Xiao-Tian Tang, Michael Longnecker, Cecilia Tamborindeguy

**Affiliations:** 1 Department of Entomology, 412 Heep Center, Texas A&M University, College station, TX 77843 USA; 2 Department of Statistics, Texas A&M University, College Station, TX USA

**Keywords:** Microbiology, Systems biology

## Abstract

‘*Candidatus* Liberibacter solanacearum’ (Lso) is a pathogen of solanaceous crops. Two haplotypes of Lso (LsoA and LsoB) are present in North America; both are transmitted by the tomato psyllid, *Bactericera cockerelli* (Šulc), in a circulative and propagative manner and cause damaging plant diseases (e.g. Zebra chip in potatoes). In this study, we investigated the acquisition and transmission of LsoA or LsoB by the tomato psyllid. We quantified the titer of Lso haplotype A and B in adult psyllid guts after several acquisition access periods (AAPs). We also performed sequential inoculation of tomato plants by adult psyllids following a 7-day AAP and compared the transmission of each Lso haplotype. The results indicated that LsoB population increased faster in the psyllid gut than LsoA. Further, LsoB population plateaued after 12 days, while LsoA population increased slowly during the 16 day-period evaluated. Additionally, LsoB had a shorter latent period and higher transmission rate than LsoA following a 7 day-AAP: LsoB was first transmitted by the adult psyllids between 17 and 21 days following the beginning of the AAP, while LsoA was first transmitted between 21 and 25 days after the beginning of the AAP. Overall, our data suggest that the two Lso haplotypes have distinct acquisition and transmission rates. The information provided in this study will improve our understanding of the biology of Lso acquisition and transmission as well as its relationship with the tomato psyllid at the gut interface.

## Introduction

‘*Candidatus* Liberibacter solanacearum’ (Lso) is a phloem-limited, Gram-negative and unculturable bacterium associated with severe plant diseases. To date, several haplotypes of this pathogen have been identified^1–6^. Haplotypes A and B infect solanaceous crops in North America and cause damaging diseases including zebra chip in potato^7,8^. These two haplotypes are transmitted by the tomato psyllid (also known as the potato psyllid), *Bactericera cockerelli* (Šulc) (Hemiptera: Triozidae). Currently, insecticides are used to control the psyllid populations and therefore Lso-related diseases because no commercially acceptable genetic resistance has been identified in the affected crops. However, the success of this strategy is limited and novel control approaches such as pathogen transmission disruption are urgently needed. The major limitations to develop these novel strategies are the complexity of the pathogen-vector systems, the lack of fundamental knowledge of the vector biology, and the fastidious nature of the pathogens.

Lso is transmitted by psyllids in a circulative and propagative manner^9–12^. Therefore, the midgut is the first psyllid organ that the bacterial pathogen colonizes and provides an essential link for understanding the biology of Lso acquisition or transmission within the tomato psyllid. More importantly, the vector gut can be a barrier for pathogen transmission^13–15^, and manipulating the interaction between the vector gut and the pathogen could be a promising way to disrupt Lso transmission. However, little is known about the acquisition or transmission of Lso at the gut interface.

Some studies focused on Lso acquisition and transmission by the tomato psyllid. For example, the transmission efficiency of Lso and the inoculation access period (IAP) required for tomato psyllid nymphs and adults to inoculate potato plants were assessed^16^. It was found that nymphs were less efficient than adults at transmitting Lso; in addition, exposure of a plant to 20 adult tomato psyllids for a period as short as 1 h resulted in zebra chip symptom development in potatoes. It was also shown that a single tomato psyllid adult was capable of inoculating Lso to potato plants within a period as short as 6 h. Lso acquisition rate by adult psyllids following different acquisition access periods (AAPs) on potato and tomato plants was also investigated^11^. It was determined that the increase of Lso titer in whole insects reached a plateau after an average of 15 days following 24- and 72-h AAPs on potato or tomato. Later, the same research group found that Lso copy numbers in psyllids peaked 2 weeks after the initial pathogen acquisition, and psyllids were capable of transmitting Lso to non-infected host plants only after a 2-week incubation period even with a short AAP of 24 h^12^.

However, the main limitation of the Lso acquisition and transmission studies is that they were carried out using double infected (LsoA and LsoB) or LsoA-infected psyllids^17,18^. Importantly, distinct acquisition or transmission could exist between the Lso haplotypes A and B. Indeed, results from our previous studies indicate that there are differences in pathogenicity between these two haplotypes in association with their host plants and insect vector, in all cases LsoB was found to be more pathogenic^19–21^.

Therefore, it is imperative to understand the biology and mode of acquisition and transmission of these two haplotypes by their psyllid vector. In the present study, our primary goal was to investigate the acquisition and transmission of Lso haplotype A and haplotype B by tomato psyllids. Toward this end, first, the accumulation of LsoA and LsoB in the gut of adult psyllids over time was quantified by quantitative real-time PCR (qPCR) and observed by immunolocalization. Second, we determined the transmission rate of each Lso haplotype and their respective latent periods. Therefore, in this study we assessed whether differences in the acquisition, latent period or transmission efficiency between the two Lso haplotypes existed. The data from this study will be the foundation to investigate the molecular interactions that occur between the insect vector and the bacterial pathogen during acquisition resulting in the development of novel strategies to disrupt Lso transmission.

## Results and discussion

### LsoA and LsoB quantification in the gut of adult psyllid

Previously, differences in the interaction of LsoA and LsoB with the tomato psyllid were reported^20^. However, the acquisition and transmission of the different haplotypes were not compared. The psyllid midgut is the first organ Lso encounter once ingested from an infected plant. Therefore, colonization of this organ is essential for Lso transmission by the psyllid vector. We first examined the acquisition of Lso haplotypes A and B by adult tomato psyllids at the gut interface.

**Figure 1:**
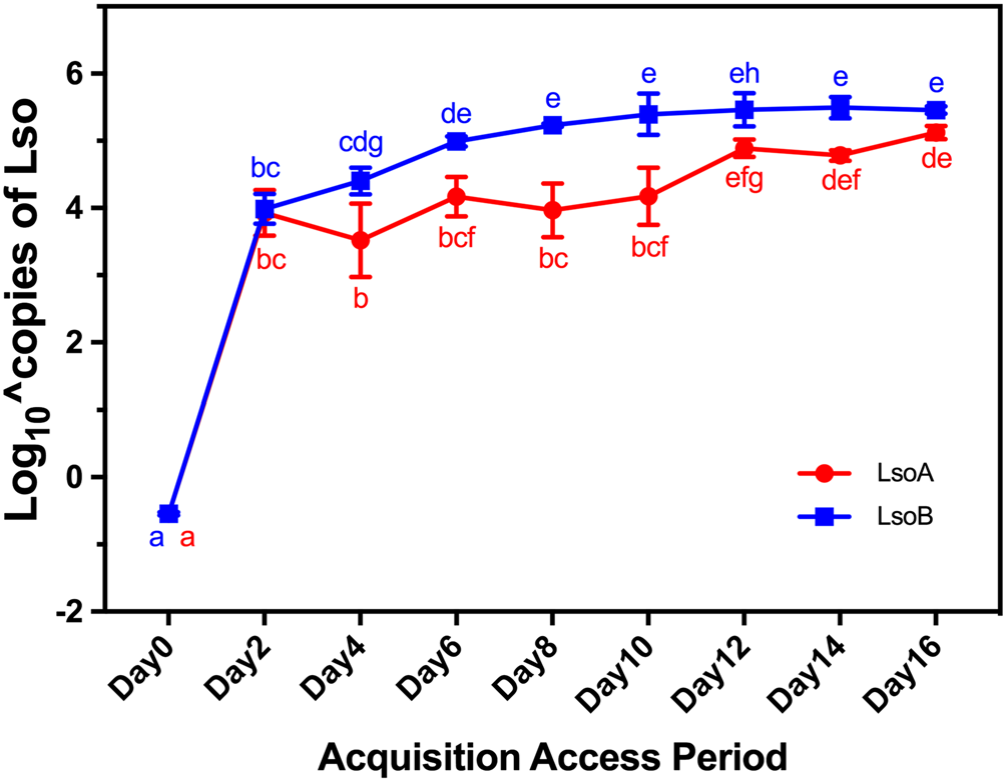
Quantification analysis of Lso copies in the gut of tomato psyllids following Lso acquisition. LsoA (red) and LsoB (blue) titer in pools of 50 guts following a 0- to 16-day acquisition access period (AAP). Data represent means ± SD of three independent replicates. Different letters indicate statistical differences at *P* < 0.05 using two-way ANOVA with Tukey's post hoc test.

A two-way ANOVA was run to examine the effect of Lso haplotype and AAP on the number of Lso copies in the psyllid midguts. There was a significant effect of the haplotype (F(1, 53) = 90.096, p < 0.01) and of the AAP (F(8, 53) = 307.656 , p < 0.01) on the number of Lso copies in the psyllid gut. The interaction of Lso haplotype and AAP on the number of Lso copies was also significant (F(8, 53) = 4.914, p < 0.001). Quantification of each Lso haplotype following different exposure times to infected plants revealed that both LsoA and LsoB populations increased continuously in the tomato psyllid guts (Fig. 1). LsoB titer increased rapidly; there were an average of 99,205 copies after 6 days of AAP, and on average a maximum of 326,214 copies was reached after 14 days of AAP. Post hoc analyses revealed there were significantly more LsoB copies after 8 days of AAP than after a 2- or 4-day AAP (*P* < 0.05). In contrast, the increase in LsoA copy number was slower after day 2, reaching on average a maximum of 135,328 copies after 16 days of AAP. After 12 days of AAP, there were significantly more LsoA bacteria than after a 2- or 4-day AAP (*P* < 0.05). In addition, between 4- and 10-day AAP, there were significantly more LsoB copies than LsoA (*P* < 0.05), while after 12-day AAP, there was no significant difference between them (*P* > 0.05). Therefore, it is likely that there is an approximately 8 day-lag for LsoA accumulation in the gut of the tomato psyllid compared to LsoB.

**Figure 2:**
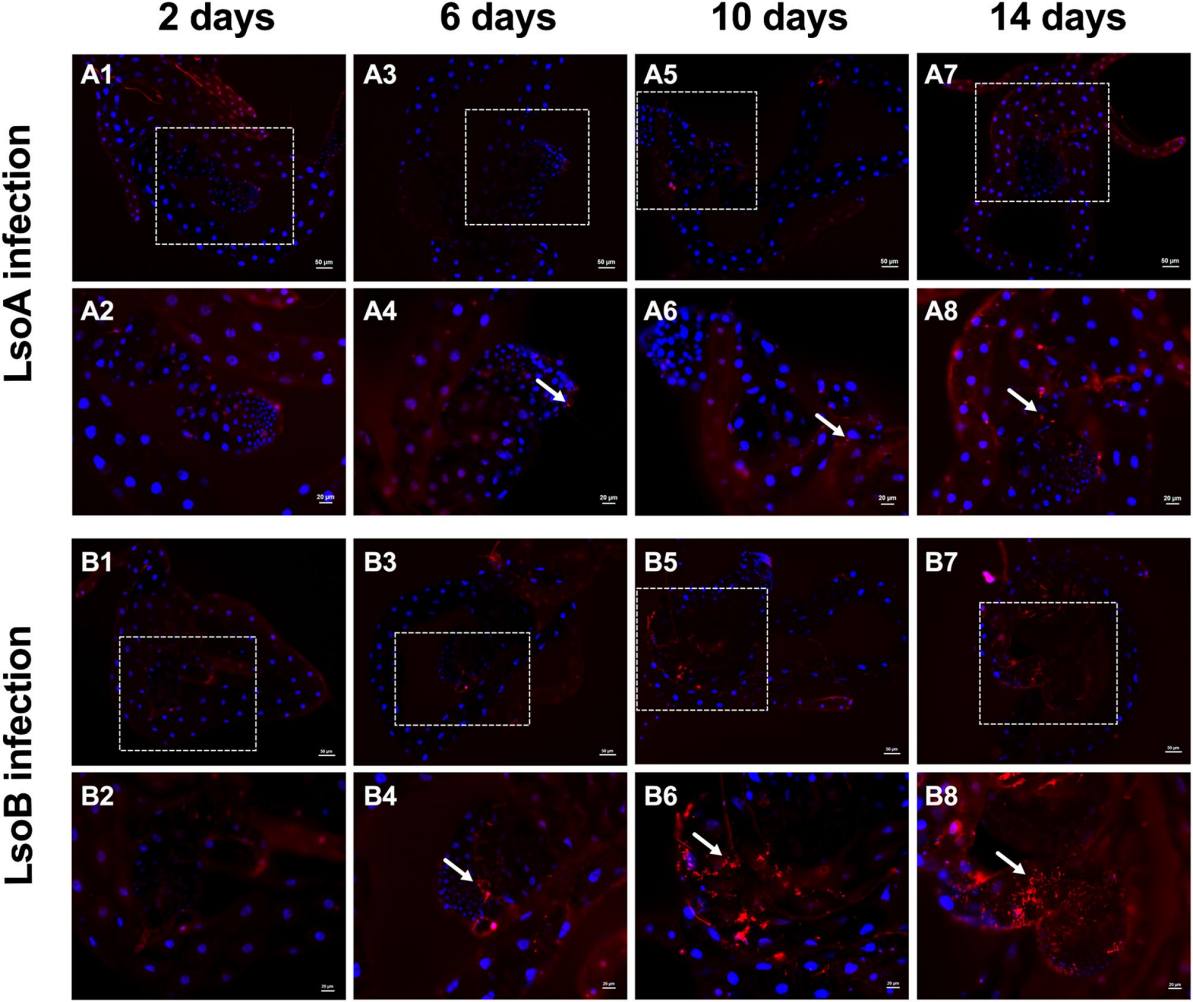
Immuno-staining of LsoA and LsoB in the guts of tomato psyllids following continuous acquisition. (**A1**,**A2**) and (**B1**,**B2**) 2 days acquisition; (**A3**,**A4**) and (**B3**,**B4**) 6 days acquisition; (**A5**,**A6**) and (**B5**,**B6**) 10 days acquisition; (**A7**,**A8**) and (**B7**,**B8**) 14 days acquisition. The white dashed rectangle indicates the enlargement region (**A2**,**A4**,**A6**,**A8**,**B2**,**B4**,**B6**,**B8**) of the filter chamber and midgut. White arrows indicate the Lso signals.

Immunofluorescence of Lso further confirmed the progressive accumulation of the bacteria in the gut. In accordance with the quantification analysis by qPCR, weak LsoA-derived signal was observed in the psyllid gut within and after 10 days of AAP (Fig. 2A1–A6), while more LsoA-derived signal was observed after 14 days of AAP (Fig. 2A7,A8). A greater amount of LsoB-derived signal was observed shortly after the beginning of the AAP, although the signal was low until 6 days of AAP (Fig. 2B1–B4). However, the LsoB-derived signal was strong and more widespread after 10 days and 14 days of AAP (Fig. 2B5–B8). Therefore, although both Lso haplotypes A and B are transmitted by the same vector, differences in acquisition were identified in the gut of adult tomato psyllids.

Overall, the infection of the adult gut by LsoA or LsoB can be divided into two stages. LsoB titer in the gut of psyllids increases rapidly and plateaus after 6 days, while LsoA accumulation is slower between days 2 and 12. Overall, these findings are consistent with the previous report^12^, which determined that the copy numbers of Lso (co-infections of LsoA and LsoB) in psyllids increased from week 0 to week 2 of the incubation period before reaching a plateau. In addition, similar propagative accumulation patterns have been reported in other plant-pathogen-insect systems; for instance, the *Rice gall dwarf virus* (RGDV) exhibits a progressive accumulation in the gut of the rice leafhopper *Recilia dorsalis* with a peak around 12 days following the initial infection^22^.

Previously, it was demonstrated that differences in Lso titer in inoculum plants do not result in differences in Lso titer in psyllids^11^. Furthermore, we have previously investigated the distribution of LsoA and LsoB titers in tomato plants^19^. The results presented here were obtained from three independent replicates, and all plants used had high Lso titers. Therefore, it is unlikely that differences in Lso titer in the infected plants significantly affected the LsoA and LsoB accumulation in psyllids. However, the differences of LsoA and LsoB titer in the gut of adult psyllids could be the result of differences of bacterial pathogenicity or the elicited psyllid immune responses. Indeed, we have previously shown that LsoB is more pathogenic than LsoA^19–21^. Similarly, the vector immune response could be one factor explaining the different Lso accumulation. For instance, autophagy is induced by RGDV in the gut of its vector, the rice leafhopper *R. dorsalis*^22^. Further investigation demonstrated that autophagy activation improves the acquisition of RGDV, whereas inhibiting the autophagy pathway reduces viral titers in the leafhopper gut. Indeed, one potential explanation for the different acquisition of LsoA or LsoB could be that distinct immune responses are elicited by each haplotype. These immune responses could affect the replication rates of Lso in the tomato psyllid gut. If indeed, different responses are elicited by each haplotype, it is possible that the response only affects Lso accumulation upon infection or in adult psyllids, because, adults that acquired Lso as nymphs have similar LsoA or LsoB titers in their guts^23^.

### Transmission of LsoA and LsoB

**Figure 3:**
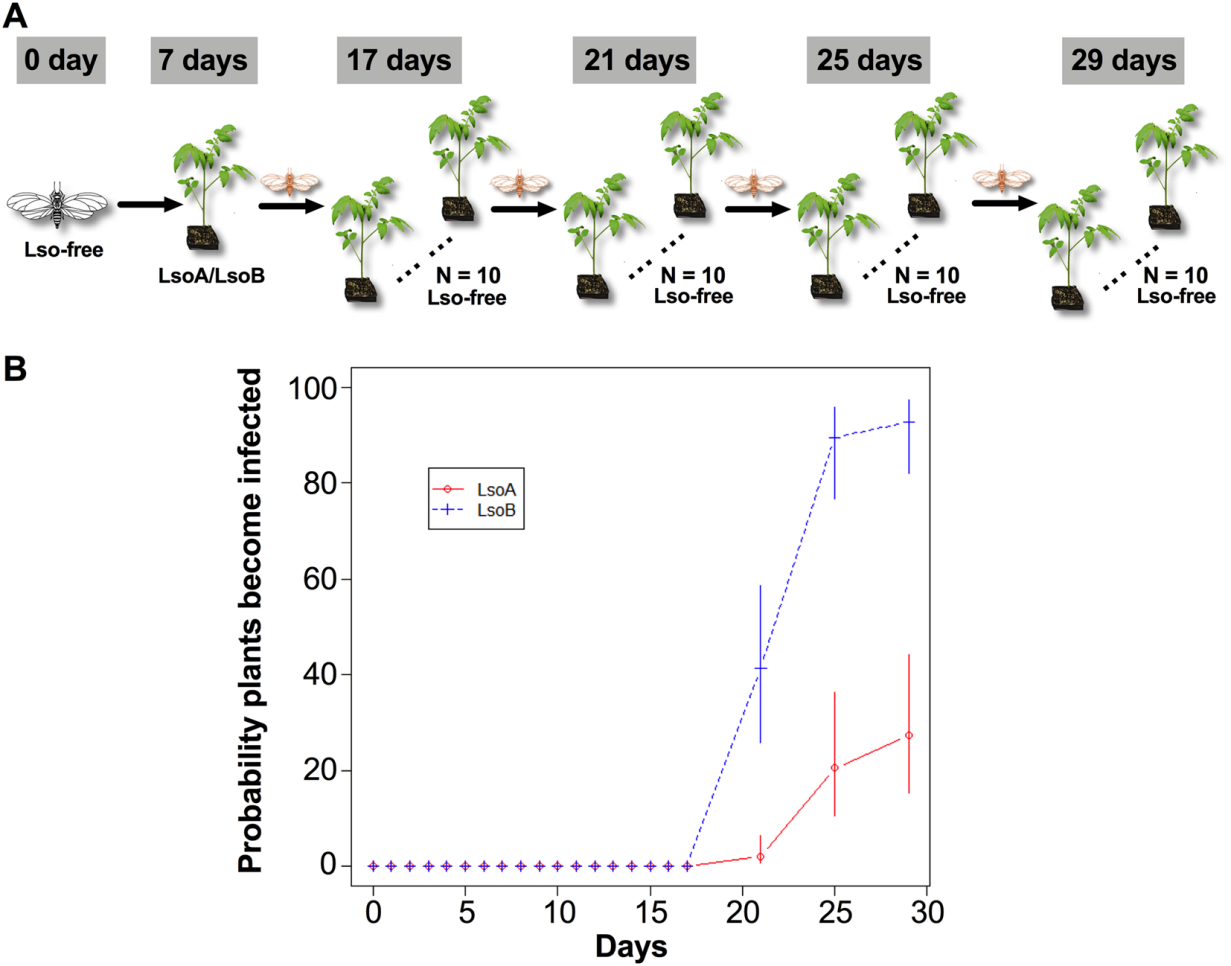
Sequential transmission test of LsoA and LsoB to tomato by psyllids after a 7-day AAP. (**A**) Adult psyllids were given a 7-day AAP on LsoA or LsoB infected tomato plants. Then, groups of five adult psyllids were transferred to 10 recipient non-infected tomato plants for a 10-day inoculation access period, and each group was sequentially transferred to a new uninfected recipient plant every 4 days as shown in the schematic representation. The days showed in the figure indicate the days post acquisition. Day 0 is the initial day Lso-free psyllids were exposed to Lso-infected plants. After 4 weeks, the presence of Lso was tested in each plant (top-tier leaves) by PCR. Psyllid and tomato plant diagrams were made by Dr. Ordom Huot. (**B**) Probability of infected plants versus days with 95% confidence interval (CI) based on logistic regression model. The latent period for LsoB in adult tomato psyllids is between 17 and 21 days, while for LsoA is between 21 and 25 days.

Because the colonization of the psyllid gut by LsoA and the increase of its titer were slower than LsoB, we hypothesized that the circulation of LsoA within the vector could also take longer than that of LsoB. Therefore, we performed sequential inoculation of tomato plants following a 7-day AAP. All the recipient tomato plants were tested Lso-negative at day 17 post acquisition. LsoB was detectable in plants as early as 21 days post acquisition with an average transmission rate to tomato plants of 43.3%. This rate increased and remained around 90% after day 25 post acquisition. In contrast, LsoA could not be detected until 25 days post acquisition, when an average of 20% of the plants tested positive for Lso infection. At 29 days post acquisition, only 30% of plants were infected by LsoA (Fig. 3). The sequential transmissions were stopped at day 29 because of psyllid mortality. As a control, on average 94.4% and 100% of the tested plants were infected by insects from the LsoA- and LsoB-infected colonies, respectively. In addition, based on the logistic regression model, there is significance of the effects of two factors (two Lso haplotypes and the days post acquisition) on the probability that a plant would become infected (*P* < 0.0001). However, no significance of the haplotype by days interaction was observed (*P* = 0.8740), indicating that the difference in the probability of infection for LsoA and LsoB remained relatively the same for the 3 days post acquisition (21, 25, and 29 days). Overall, haplotype LsoB had significantly higher odds (probability of infection) across the 3 days post acquisition relative to LsoA (Fig. 3). We also observed a lag between LsoA and LsoB transmission, which is consistent with the differences of Lso accumulation in the gut obtained by quantification. Therefore, LsoB had a shorter latent period than LsoA. Specifically, the latent period of LsoB was between 17 and 21 days, while LsoA took between 21 and 25 days to be transmitted. The latent period for Lso transmission observed here is consistent with the previous study which determined that a 2-week latent period was required for adult tomato psyllids to transmit Lso following a 24- or 72-h AAP^12^. While the authors did not test transmission of each Lso haplotype separately, they did not observe significant differences of latent period between the two Lso haplotypes^12^. The discrepancies between those results and ours could result from the analysis of the transmission of each Lso haplotype separately. Indeed, it is possible that competition or cooperation between the Lso haplotypes occurs within the insect or the host plant. Another difference between the two studies was the use of tomato as source and recipient hosts in the present study, while before potato was used for the acquisition and transmission analyses^12^. Additionally, there are four tomato psyllid haplotypes in North America^24,25^, and the previous study used central haplotype psyllids^12^, our study was performed with western haplotype psyllids. Therefore, it is possible that genetic differences among the tomato psyllid populations or even the endosymbionts associated with each population^26^ result in different capacity for Lso transmission. Indeed, it appears that the inoculation rates of the bacterial pathogen ‘*Ca.* L. asiaticus’ (CLas) by adult Asian citrus psyllid populations in North America might be less efficient than those previously reported for the vector’s Chinese populations^27,28^.

Several factors could potentially account for the difference of LsoA or LsoB transmission rates by tomato psyllids. First, the ability of Lso to infect the gut or to cross from the gut into the hemocoel can determine its transmission efficiency. Indeed, as discussed above the insect immune system may prevent or delay the pathogen circulation through the vector. For instance, an apoptotic immune response was observed in the gut of Asian citrus psyllid adults from the CLas-infected colonies*,* but not in the nymphal gut^29,30^. It is probable that the apoptotic response serves to limit the acquisition or transmission efficiency of CLas by the Asian citrus psyllid. Indeed, CLas titer increased at a faster rate when the bacterium was acquired by nymphs compared to adults^31^. Consequently, future analyses should focus on the immune responses elicited by each Lso haplotype on the psyllid vector. In addition, LsoB was found to be more pathogenic in association with their host plant and insect vector^19–21^. It is therefore possible that LsoB is able to better defend itself against the psyllid immune response, or even manipulate those defenses to its advantage^32^. As a consequence, LsoB could be acquired and transmitted with higher efficiency than LsoA. Second, the salivary gland is another potential barrier for Lso transmission. It was established previously that over 10,000 Lso genomes were required in the salivary glands for tomato psyllids to effectively transmit the bacterium^12^. Based on our results, we speculate that LsoB reaches that threshold earlier than LsoA. This could be because LsoB is able cross the gut barrier faster than LsoA, or that LsoB required less time to colonize the salivary glands and multiply, reaching the transmission threshold earlier.

This study revealed that LsoB can be efficiently acquired and transmitted by adult tomato psyllids, however, transmission of LsoA by psyllids that acquire the pathogen during adulthood is less efficient. The epidemiological implications if any, of this difference in transmission efficiency between the two Lso haplotypes need to be investigated.

In summary, we suggest the existence of differences in the acquisition and transmission of the Lso haplotypes A and B. LsoB titer increased more rapidly in the adult psyllid gut than LsoA. Furthermore, LsoB had a significantly higher transmission efficiency by adult psyllids than LsoA and a shorter latent period between acquisition and inoculation. The information in the present study could be of significance for disease epidemiology and to develop strategies to disrupt pathogen transmission. Additional studies are needed to explore the accumulation of the two Lso haplotypes in the salivary glands and determine the Lso transmission by other psyllid haplotypes.

## Materials and methods

### Insect colonies and tomato plants

Lso-free, LsoA-, and LsoB-infected psyllid colonies (western haplotype) were maintained separately on tomato plants in insect cages (24 × 13.5 × 13.5 cm, BioQuip, Compton, CA) at room temperature 24 ± 1 °C and photoperiod of 16:8 h (L:D)^33^.

To obtain Lso-infected tomato plants, tomato plants variety Moneymaker, were grown from seeds (Victory seed company, Molalla, OR). Six-week-old tomato plants were infected as described previously^34^ using three male psyllids harboring LsoA or LsoB, respectively. After 1 week, all psyllids were removed from the tomato plants. Three weeks after Lso inoculation, the plants (top-tier leaves) were tested for Lso infection using the LsoF/OI2 primers^35^ and the Lso haplotype in the plants was confirmed using the Lso SSR-1 primers^3^.

### Psyllid exposure to Lso and gut dissection

Young Lso-free adult psyllids (less than 7 days old, a mix of males and females) were transferred to LsoA- or LsoB-infected tomato plants. To minimize the effect of differences in Lso titer within infected plants^19^, groups of over 60 adult psyllids were caged on leaves at the same level of the tomato plants using organza bags. Every 2 days, 50 insects from a same group were collected in order to obtain 2-, 4-, 6-, 8-, 10-, 12-, 14-, and 16-day Lso-exposed psyllids. The groups of 50 psyllids were randomly caged on different plants. The experiments were stopped after 16 days of AAP because adult tomato psyllids live on average 1 month and high mortality of psyllids started to be observed.

Guts from the LsoA- or LsoB-exposed psyllids were dissected under the dissecting microscope^36^. DNA from pools of 50 guts was purified following the protocol of blood/tissue DNA extraction kit (Qiagen, Hilden, Germany); each pool was used as an individual template for qPCR analysis. Thus, each pool of 50 guts represented one replicate, and there were three replicates for each combination of exposure time point and haplotype. Each replicate was obtained by using independent LsoA- or LsoB-infected plants as Lso inoculum. All the experiments were conducted at 24 ± 1 °C.

### Quantification of Lso

The Lso 16S rDNA specific primers (LsoF: 5′-CGAGCGCTTATTTTTAATAGGAGC-3′ and HLBR: 5′-GCGTTATCCCGTAGAAAAAGGTAG-3′)^35,37^ were used for Lso quantification in the guts of adult psyllids, and the psyllid 28S rDNA primers (28S rDNAF: 5′-AGTTTCGTGTCGGGTGGAC-3′ and 28S rDNAR: 5′-AACATCACGCCCGAAGAC-3′)^34^ were used as internal control. qPCR was performed using SYBR Green Supermix Kit (Bioline, Taunton, MA) according to manufacturer’s instructions. Each reaction contained 25 ng of DNA, 250 nM of each primer, and 1X of SYBR Green Master Mix; the volume was adjusted with nuclease-free water to 10 μL. The qPCR program was 95 °C for 2 min followed by 40 cycles at 95 °C for 5 s and 60 °C for 30 s. The qPCR assays were performed using a QuantStudio 6 Flex Real-Time PCR System (Applied Biosystems, Foster City, CA). Reactions for all samples were performed in triplicates with a negative control in each run. In order to standardize the amount of Lso in psyllid guts, data are reported as delta Ct = (Ct of Lso gene) − (Ct of psyllid 28S gene). The biological replicates were analyzed and the average delta Ct value was used to quantify the levels of Lso. A standard curve was prepared for quantification of Lso in psyllid guts using a plasmid containing the Lso 16S rDNA target. For the standard curve, 10-fold serial dilutions of the plasmid were performed. The procedure for the standard curve preparation and the calculations of molecules (copies) followed the methods described in^38^ and^34^. The Lso copy number in each sample was estimated by comparing delta Ct values of each sample to the standard curve.

### Immunolocalization of Lso in psyllid guts

Immunolocalization was used to visualize Lso in the Lso-exposed psyllid gut tissues. The psyllid guts were dissected in 1X phosphate buffered saline (PBS) (Sigma-Aldrich, St. Louis, MO) from adults at 4-day-infection intervals. Then, the guts were fixed in 4% paraformaldehyde for 30 min at room temperature. After fixation, the guts were incubated with Sudan Black B (Sigma-Aldrich) for 20 min to quench autofluorescence as described in^39^. Next, the guts were permeabilized by adding 0.1% Triton X-100 (Calbiochem/EMD Chemicals, Gibbstown, NJ) for 30 min at room temperature, and washed three times with PBS containing 0.05% Tween 20 (PBST) prior to a 1-h blocking incubation at room temperature with blocking buffer (PBST with 1% [w/v] bovine serum albumin). Lso immunolocalization was performed using a rabbit-derived polyclonal antibody (GenScript Corp, Piscataway, NJ) directed against Lso OMP-B (Ab-OMP-B) which detects both Lso haplotypes^23^. The guts were incubated with the antibody (diluted 1:500) overnight at 4 °C. The guts were then washed three times with PBST and incubated with an Alexa Fluor 594 goat anti-rabbit IgG secondary antibody (diluted 1: 2000; Invitrogen, Carlsbad, CA) for 1 h at room temperature. The guts were washed again three times with PBST, and mounted with one drop of Vectashield mounting medium with 4′,6-diamidino-2-phenylindole (Vector Laboratories Inc., Burlingame, CA) on a microscope slide. The slide was covered with a glass coverslip and sealed with nail polish. The guts were examined using Axioimager A1 microscope (Carl Zeiss microimaging, Thornwood, NY, USA) and the images were collected and analyzed with the Axiovision Release 4.8 software (Carl Zeiss).

### Lso transmission assay

The sketch of transmission assay is shown in Fig. 3. Young Lso-free adult psyllids (less than 7 days old, mix of males and females) were exposed to LsoA- or LsoB-infected plants for a 7-day AAP as described above. Then, groups of five LsoA- or LsoB- exposed psyllids were transferred to ten 4-week-old non-infected recipient tomato plants for a 10-day IAP. Then every 4 days, the same batch of LsoA- or LsoB-exposed psyllids were continuously transferred to new set of ten non-infected recipient tomato plants. In total, four rounds of transfer were conducted. As a positive control, we also transferred adult psyllids from the LsoA- or LsoB-infected colonies, which acquire Lso as nymphs, to six 4-week-old non-infected recipient tomato plants. Four weeks after the end of the IAP, the plants (top-tier leaves) were tested for Lso infection as described above. The transmission assays were performed three times.

### Data analysis

The data were analyzed using R (https://www.r-project.org/) and SAS (Ver. 9.4). For the analysis of Lso genome copies in the gut of psyllid, the raw data typically have a Poisson distribution. Thus, first a log10-transformation was applied to the data and then analyzed using a two-way ANOVA with Tukey’s post hoc test. Homogeneity of variance was verified with the Levene's Test, and the residuals' normal distribution was verified using the Shapiro–Wilk normality test. The percentage of infected plants from the three replicated experiments of each time point were determined using a logistic regression model, which was fit to evaluate the effects of two factors (two Lso haplotypes and the days post acquisition) on the probability that a plant would become infected. The log-odds model is given by:

$${\text{Log}}[{\text{p/}}(1 - {\text{p}})] \, = {\text{ b}}0 \, + {\text{ b}}1\;{\text{ Haplotypes }} + {\text{ b}}2\;{\text{ Days }} + {\text{ b}}3 \, \;{\text{Haplotypes}}*{\text{Days }} + {\text{ e}},$$

where p = probability plant becomes infected after being exposed to the Lso haplotypes a given number of days after the potato psyllid was exposed to the Lso haplotypes and p/(1 − p) is the odds of an exposed plant becoming infected.
